# Impact of Rotavirus Vaccination on Hospitalisations in Belgium: Comparing Model Predictions with Observed Data

**DOI:** 10.1371/journal.pone.0053864

**Published:** 2013-01-18

**Authors:** Baudouin Standaert, Jorge A. Gomez, Marc Raes, Serge Debrus, F. Raúl Velázquez, Maarten J. Postma

**Affiliations:** 1 Health Economics, GlaxoSmithKline Vaccines, Wavre, Belgium; 2 Medical Department, GlaxoSmithKline Vaccines Latin America, Victoria, Buenos Aires, Argentina; 3 Department of Pediatrics, Jessa Hospital, Hasselt, Belgium; 4 Vaccine Development and Discovery, GlaxoSmithKline Vaccines, Wavre, Belgium; 5 Medical Research Unit on Infectious Diseases, Pediatrics Hospital, National Medical Center-Century XXI, Mexican Institute of Social Security (IMSS), Mexico City, Mexico; 6 Unit of Pharmacoepidemiology and Pharmacoeconomics (PE2), Department of Pharmacy, University of Groningen, Groningen, The Netherlands; Johns Hopkins Bloomberg School of Public Health, United States of America

## Abstract

**Background:**

Published economic assessments of rotavirus vaccination typically use modelling, mainly static Markov cohort models with birth cohorts followed up to the age of 5 years. Rotavirus vaccination has now been available for several years in some countries, and data have been collected to evaluate the real-world impact of vaccination on rotavirus hospitalisations. This study compared the economic impact of vaccination between model estimates and observed data on disease-specific hospitalisation reductions in a country for which both modelled and observed datasets exist (Belgium).

**Methods:**

A previously published Markov cohort model estimated the impact of rotavirus vaccination on the number of rotavirus hospitalisations in children aged <5 years in Belgium using vaccine efficacy data from clinical development trials. Data on the number of rotavirus-positive gastroenteritis hospitalisations in children aged <5 years between 1 June 2004 and 31 May 2006 (pre-vaccination study period) or 1 June 2007 to 31 May 2010 (post-vaccination study period) were analysed from nine hospitals in Belgium and compared with the modelled estimates.

**Results:**

The model predicted a smaller decrease in hospitalisations over time, mainly explained by two factors. First, the observed data indicated indirect vaccine protection in children too old or too young for vaccination. This herd effect is difficult to capture in static Markov cohort models and therefore was not included in the model. Second, the model included a ‘waning’ effect, i.e. reduced vaccine effectiveness over time. The observed data suggested this waning effect did not occur during that period, and so the model systematically underestimated vaccine effectiveness during the first 4 years after vaccine implementation.

**Conclusions:**

Model predictions underestimated the direct medical economic value of rotavirus vaccination during the first 4 years of vaccination by approximately 10% when assessing hospitalisation rates as compared with observed data in Belgium.

## Introduction

The economic assessment of the newer rotavirus vaccines (*Rotarix*® [Rotarix is a registered trade mark of the GlaxoSmithKline group of companies] and *Rotateq*™ [Rotateq is a trademark of Merck & Co. Inc.]) at the time of their first introduction in 2006 was largely model-based, in the absence of long-term data on vaccine effects [1]–[3]. Most assessments at that time used static Markov cohort models instead of dynamic models [4], which simplified the model construction, the number of assumptions introduced, and the data requirements [5]. Cohort models analyse the vaccine situation at epidemiological steady-state [6] when vaccination is already well established in the population at risk, children less than 5 years old in the case of rotavirus. More recently, there has been a shift towards developing more complex models for estimating the total benefit of rotavirus vaccines because a herd effect after vaccination has been reported from observational data [7]–[10].

Observational studies have shown that rotavirus infection produces partial immunity after each exposure [11], [12], with complete immunity acquired after three to four infections. This partly explains the peculiar distribution of rotavirus disease as a function of age, which forms a bell-shaped curve during the first two years of the birth cohort. A Markov cohort model can replicate the natural history of rotavirus disease in a birth cohort over time, with the highest disease burden occurring in children aged between 6 months and 2 years, followed by a sharp decline up to the age of 5 years, after which natural immunity across the cohort is maintained.

The early economic models of rotavirus vaccination included much uncertainty due to the many unknowns in the data available at the time, such as the impact of rotavirus disease on quality-adjusted life-years (QALY), waning of vaccine efficacy over time (presumed from clinical trials), and the proportion of rotavirus gastroenteritis cases who do not seek medical care [13]. Such unknowns were modelled using ‘best-guess’ baseline assumptions, tested in sensitivity analyses to evaluate their impact on the incremental cost-effectiveness ratio (ICER).

Among these unknowns, vaccine waning is of particular interest. Vaccine efficacy in the cohort models was derived from clinical trial results for the rotavirus vaccines. The trials indicated higher vaccine efficacy against rotavirus diarrhoea during the first year than in subsequent years [14]. However, it should be noted that the decrease in vaccine efficacy measured over time in the European trial was mainly due to a large reduction in rotavirus diarrhoea events reported in the non-vaccinated arm (−42%), rather than due to an increase in the numbers of events in the vaccinated arm as one would expect from vaccine waning over time. This indicated that the vaccine waning assumption in the early models should be re-examined.

The two rotavirus vaccines have now been in use for several years, and real-life data are becoming available. A few follow-up studies after vaccine introduction provide information on real-life vaccine effectiveness on specific mortality reduction in Mexico and hospitalisation rates in Brazil, US, Australia and some European countries [15]–[22]. It is now possible to test whether the model-predicted results presented at the time of the product launch were accurate enough to report reliable cost-effectiveness data. Clearly, should substantial discrepancies occur between prediction and observation, understanding the possible causes would be valuable to improve the next generation of vaccine models. Few attempts have yet been made in the published literature to compare results predicted by models at vaccine introduction with real-life data observed over time.

Belgium provides a good opportunity to conduct such a comparison for rotavirus vaccination, as modelled estimates and observed data from a follow-up study of four years post-vaccination and two years pre-vaccination are available [18], [23]. In a previous paper on the impact of rotavirus vaccination on hospitalisation in Belgium, we reported that the observed reductions in rotavirus hospitalisations after vaccine introduction were greater than those predicted by modelling [18]. In the present analysis, we have explored this discrepancy further using the most recent data from the observational study (up to four years post-vaccination) to identify potential reasons for the differences, and have adjusted the modelled ICER for differences between predicted and observed data.

## Methods

### Model construction

When rotavirus vaccination was introduced in Belgium in 2006, a Markov cohort model, mainly based on the model published by Melliez et al. [23], [24], assessed at vaccine steady-state the rate of rotavirus acute gastroenteritis (AGE) in a birth cohort by month up to the age of 5 years. The model included different management options typical for the Belgian context such as staying at home, seeking medical advice from a primary care physician or a specialist, visiting the emergency room, or admission to hospital. The distribution of rotavirus AGE cases by age was constructed following a Weibull function [25]. A Weibull distribution with its shape (k = 1.5) and scale (λ =  24.2) parameters allows replication of the distribution of rotavirus disease as a function of age, influenced by the gradual disappearance of maternal antibodies after birth and by new rotavirus infections appearing over time that stimulate the development of natural immunity. The two parameters should be adjusted for country-specific data using calibration techniques specifying breastfeeding behaviour and the frequency of infection exposure over time.

Vaccine efficacy data used in the model were taken from a European trial, which showed a decrease in effect over time that differed between mild (staying at home), moderate (seeking medical advice), and severe (hospitalised) cases [14].

For each level of disease severity, specific costs and utility scores were applied [23]. The model compared vaccinated and unvaccinated cohorts and allowed for changes in vaccine coverage. Herd protection was not included. The model estimated the vaccine effect on the number of AGE events, medical visits, emergency visits, hospitalisations and deaths in a birth cohort of children up to the age of 5 years. It also reported the overall cost, QALY impact, and ICER for vaccination compared with no vaccination.

### Observational study

A vaccine impact study was set up one year after the introduction of the rotavirus vaccine in Belgium [18], [21]. Full details and the results for the first two years post-vaccination (up to May 2009) have been published elsewhere [18]. Data were collected retrospectively after each rotavirus season from a sample of 12 Belgian hospitals. All children aged ≤5 years who had a rotavirus detection test performed at a participating hospital from 1 June 2004 to 31 May 2006 (pre-vaccination study period) or 1 June 2007 to 31 May 2010 (post-vaccination study period) were eligible. Only hospitalised children were included, and data were analysed for the nine centres with a complete dataset. Ethical approval was not required because there was no medical file consultation.

The post-vaccination study period was divided into successive years, each running from June to May (June 2007–May 2008, June 2008–May 2009 and June 2009–May 2010) to cover the winter rotavirus season. The period between 1 June 2006 and 31 May 2007 was not included in our study, because reimbursement for rotavirus vaccination was not available for the whole of this period (partial reimbursement was introduced in Belgium in November 2006 for *Rotarix*® and in June 2007 for *RotaTeq*™ [18]). Thus, although June 2006–May 2007 could be considered as the first year post-vaccination, the date of reimbursement meant that it was neither fully pre-vaccination nor fully post-vaccination. In this study we therefore analysed data from the second post-vaccination year (June 2007–May 2008) onwards. For each year the number and the proportion of rotavirus-positive episodes were calculated per week. Hospitalisation was classified as AGE-driven if the stool sample was collected within 48 hours of hospitalisation. The most relevant variable to compare in the pre- and post-vaccination periods is the absolute number of rotavirus-positive episodes observed, assuming no change in catchment area between the study periods for each participating hospital.

### Comparison between observed and modelled data

From the raw observed data we first calculated the frequency of hospitalisation per week for each of five age groups (0–1 year; 1–2 years; 2–3 years; 3–4 years; 4–5 years) over a period of one year for the pre-vaccination period and for the second (June 2007–May 2008) and fourth (June 2009–May 2010) years post-vaccination. As the data are from a small sample, it is likely that data from a larger sample would follow a smoother distribution. This is represented by adjusting the raw frequencies to smoothed parametric curves using *@RISK* 5.7 software (Palisade Corporation, US). The software is an add-in program in Microsoft *Excel®* that uses the collected data as input variables, for which it creates a distribution expressed as a probability density function from a list of around 20 continuous parameterised distributions. Since all probability distribution functions must have a unit area, the software automatically scales the probability values so that the density curve has an area of one. The method of least squares is used to minimize the Root-Mean Square Error between the curve points and the theoretical distribution function selected (RMS Error value <0.05 or the best Chi-squared statistics noted between the observed data and selected parametric distribution). The figures obtained are referred to in this paper as smoothed curves, or adjusted observational data. Because the smoothed curves are parameterised distributions, they are easier to work with when calculating values for the areas under curves.

The original modelled data were derived from a hypothetical birth cohort followed over time from birth to age 5 years, whereas the observed data were derived from multiple one-year cross-sectional observations in a population of children aged up to 5 years. To allow a transparent comparison between the two, it was necessary to transform the results from the cohort model to a population approach, which could be compared with the population data from the observational study.

**Figure 1:**
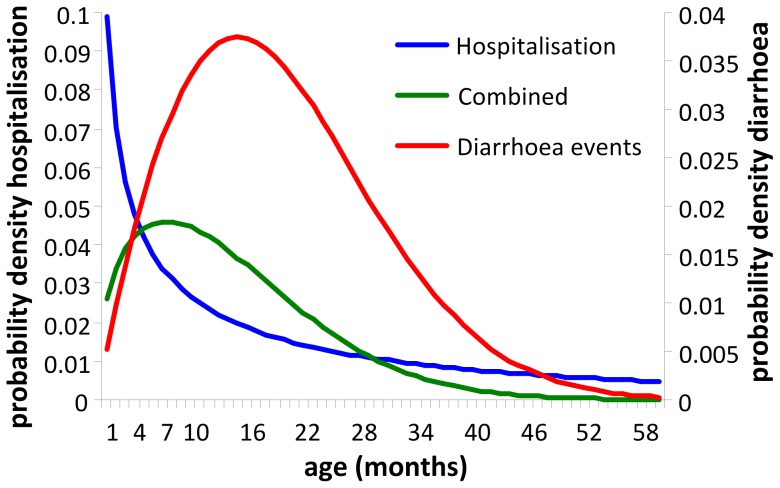
Probability density functions for defining hospitalisation rate as a function of age (pre-vaccination). Diarrhoea events, distribution of rotavirus AGE events as a function of age from birth to age 60 months (5 years); Hospitalisation, distribution of hospitalised rotavirus events from birth to age 60 months (5 years); Combined. combined function.

This transformation includes as a first step elaborating the original single cohort model into a multiple cohort model with five birth cohorts, sequencing the start by delaying each subsequent year. This construction allows the vaccine coverage rate and the vaccine efficacy to be varied by month, year, and age group. Vaccine efficacy and coverage values are shown in Table 1. The baseline age distribution for rotavirus AGE events in each cohort model followed a Weibull function as described above. The age distribution for hospitalised events used a modified distribution to take into account the higher hospitalisation rate in infants and young children. The parameter values used in each Weibull distribution are shown in Table 1. The net hospital age-distribution result in each cohort model was the combination of the two distributions, multiplying the density probability function of the AGE distribution by the hospitalisation distribution, leading to a combined distribution (Figure 1).

**Table 1:** Model-specific adaptations to fit pre-vaccination observed data.

Parameter	Value
Disease distribution as a function of age from birth to month 60	Weibull 1 with parametric distribution of k = 1.5 and λ = 24.2
Hospitalisation distribution as function of age from birth to month 60	Weibull 2 with parametric distribution of k = 0.6 and λ = 29.3
1^st^ year vaccine coverage	60%*
2^nd^ year vaccine coverage	80%*
3^rd^ year vaccine coverage	85%*
4^th^ year vaccine coverage	85%*
Estimated vaccine efficacy 1^st^ year	95%
Estimated vaccine efficacy adjustment every subsequent year post-vaccination (reduction in efficacy to represent vaccine waning)	15% per year

* reported from Intercontinental Medical Statistics (IMS) data.

The next step was to introduce two assumptions in the analysis that could be checked against the observed data. First, we assumed that the annual epidemic rotavirus spread of hospitalised disease in children aged up to 5 years followed a normal distribution. Registry data on the annual spread of rotavirus indicate that this assumption is acceptable [26]. We therefore constructed a normal distribution over a 52-week period with a standard deviation of 0.16 for a mean value of 1, by which the spread of the disease is absent over a period of 16 weeks per year. The second assumption was that the age distribution per week in the normal distribution followed the combined distribution of the age cohort, as defined in Table 1. As a consequence, the disease spread each year appeared first and disappeared last in infants and young children, compared with older children, reflecting the distribution with a higher hospitalisation rate in infants and young children.

This approach allowed precise measurement of differences between the impact of vaccination in the model construct and the observational data. Any differences identified between the observation and the modelled results were explored to see if potential explanations could be found. Once potential explanations were identified, we adjusted the model input values to be equivalent to the observed data to estimate adjusted ICERs.

## Results

**Figure 2:**
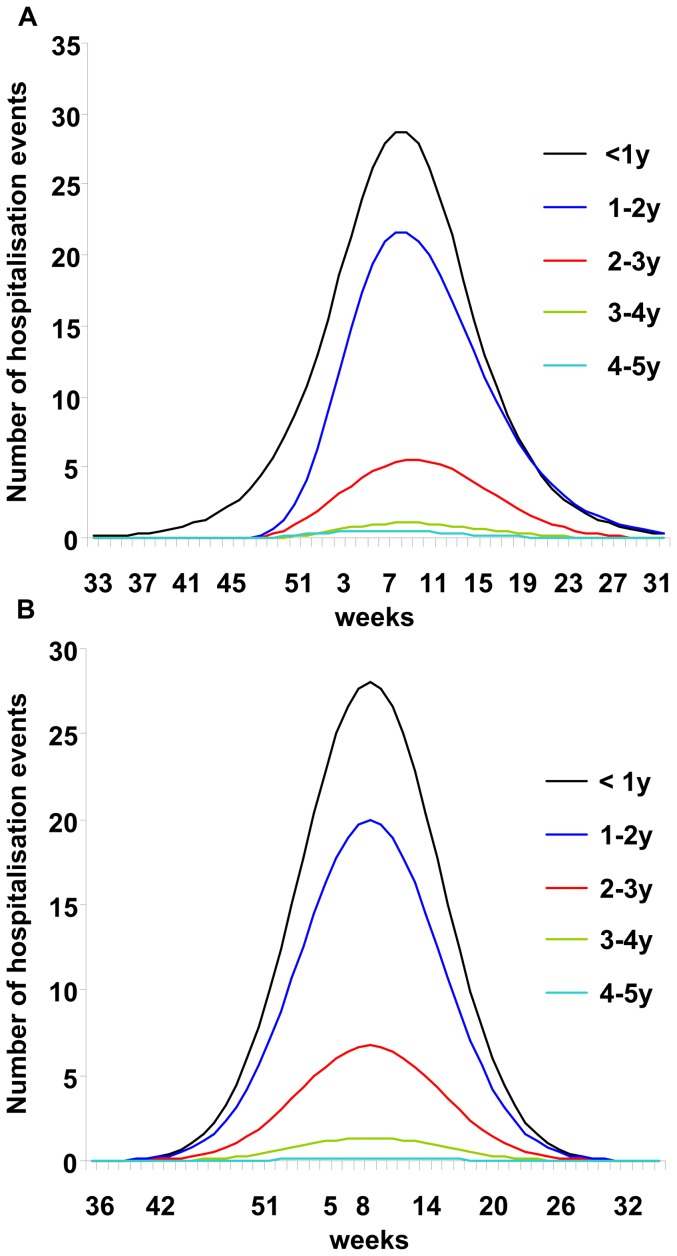
Observed and modelled numbers of hospitalised rotavirus events (pre-vaccination). Adjusted observed data (smoothed curves) by age group (A); Results by age group from the multiple age-cohort model (B). Weeks are numbered according to seasonal distribution.

Figure 2 shows the pre-vaccination curves for adjusted observed data on the number of hospitalisation events by week and age group (Figure 2A) that were similar to the modelled results from the multiple age-cohort model (Figure 2B). As expected, the pre-vaccination peak in rotavirus hospitalisations was highest in children aged <1 year. In the observed data the peak appeared at approximately the same time of the year (Week 8) in all age groups, consistent with seasonal rotavirus spread and indicating a dependency in rotavirus transmission between age groups. The two assumptions introduced into the multiple cohort model to construct a population approach appeared to hold when comparing the distribution results of the model and the observation data. Moreover, there was close agreement between the observed and modelled numbers of rotavirus hospitalisations by age group per year for the pre-vaccination scenario (Table 2).

**Table 2:** Reported hospitalisation events over one year by age group pre-vaccination, for observed and modelled data.

	Number (%) of rotavirus hospitalisations	
Age group (years)	Observed	Modelled
<1	454 (51.8%)	439 (49.8%)
1–2	319 (36.2%)	312 (35.4%)
2–3	86 (9.7%)	106 (12.0%)
3–4	15 (1.7%)	21 (2.4%)
4–5	7 (0.8%)	3 (0.3%)
**Total**	**880**	**881**

**Figure 3:**
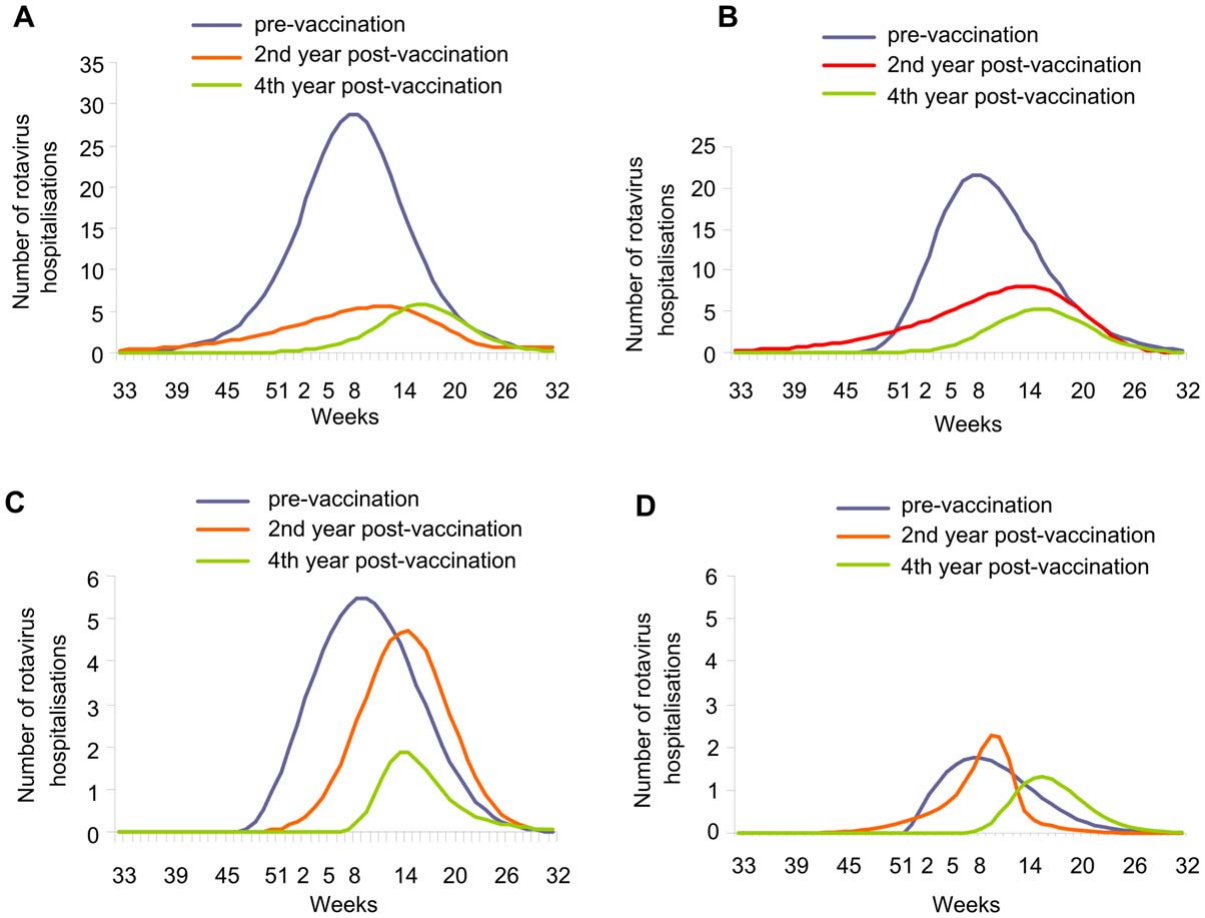
Impact of rotavirus vaccination after 2 and 4 years of vaccination by age group (observed data). Aged <1 year (A); Aged 1–2 years (B); Aged 2–3 years (C); Aged >3 years (D). Weeks are numbered according to seasonal distribution.

In the post-vaccination period, the observed data showed that the seasonal peak in rotavirus hospitalisations was reduced in magnitude and delayed (shifted to the right) in the second year after vaccine introduction for the first two age groups, with further reduction and delay in the fourth year across all age groups (Figure 3A–D).

As the observational study included children aged up to 5 years, some of the children enrolled in the post-vaccination period were too old for vaccination when the vaccine was introduced, and thus were unvaccinated. The age threshold increased in successive years post-vaccination. In the second year post-vaccination (June 2007–May 2008), the maximum age of vaccinated children was 21 months (born in or after September 2006, just in time to receive vaccination after reimbursement of the first rotavirus vaccine product in November 2006, and included in the last month of that study year in May 2008), and in the fourth year post-vaccination (June 2009–May 2010) the maximum age of vaccinated children was 45 months (born in or after September 2006 and included in the last month of that study year in May 2010). The reduction in hospitalisations post-vaccination compared with pre-vaccination observed in the age groups who were too old to be vaccinated (Table 3), indicated that the vaccine had an indirect protective effect.

**Table 3:** Observed and modelled data pre- and post-vaccination by year and age group.

		Post-vaccination			% reduction from pre-vaccination		
Age group (years)	Pre-vaccination	Year 2	Year 4	Adjusted	Year 2	Year 4	Adjusted
**Observed**							
<1	454	125	77		72%	83%	
1–2	319	164	72		49%	77%	
2–3	86	61	17		29%	80%	
3–4	15	9	10		40%	33%	
4–5	7	9	3		−29%	57%	
**Total**	**880**	**368**	**179**		**58%**	**80%**	
**Modelled**							
<1	439	146	127	127	67%	71%	71%
1–2	312	161	111	73	48%	64%	77%
2–3	106	106	48	29	0%	55%	73%
3–4	21	21	11	7	0%	48%	67%
4–5	3	3	3	3	0%	0%	0%
**Total**	**881**	**437**	**300**	**239**	**50%**	**66%**	**73%**

Number of rotavirus hospitalisations from observed and modelled data. Adjusted data refer to modelled data with vaccine waning removed from the model (i.e. assuming that vaccine efficacy is the same in subsequent years as in the first year).

There is a second group of children ineligible for vaccination, those too young to receive the vaccine (aged up to 2 months). The number of observed rotavirus gastroenteritis events in this age group also declined in the years after vaccine introduction (Table 4) (Chi-square-test for trend, p<0.01). The results indicated that a herd protection effect may also occur in children too young for vaccination, due to reduced transmission of natural rotavirus infection after vaccine introduction.

**Table 4:** Rotavirus hospitalisations pre- and post-vaccination in infants <3 months old.

	Number of rotavirus hospitalisations			
Age group	Pre-vaccination	Post-vaccination second year	Post-vaccination third year	Post-vaccination fourth year
0-1 month	18	12	4	6
1-2 month	46	8	13	11
2-3 month	38	23	14	6

(Chi-square for trend: p<0.001).

**Figure 4:**
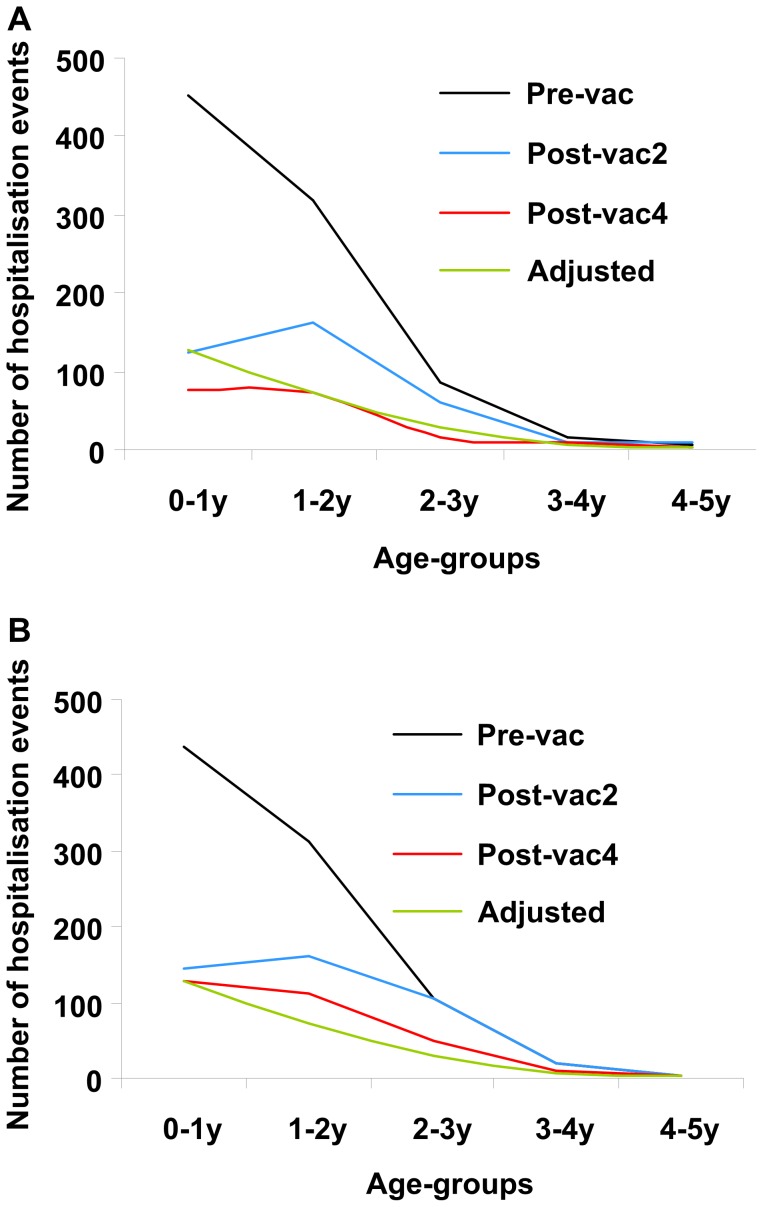
Pre- and post-vaccination data by year and age group. Observed data (A); Modelled data (B). Pre-vac, pre-vaccination; Post-vac2, second year post-vaccination; Post-vac4, fourth year post-vaccination; Adjusted, modelled results assuming no vaccine waning, included for comparison purposes.

The overall herd effect that occurred in real life was not included in the model. But the more rapid decrease in hospitalisations in the observed data, compared with the model, is also noteworthy because the model assumed a decrease in vaccine efficacy year on year (Table 1), which was not apparent in the observed data. In sensitivity analysis, the model was run with no decrease in vaccine efficacy (i.e. assuming that vaccine efficacy was the same in subsequent years as in the first year). These data are shown in Table 3 and Figure 4 as ‘Adjusted’ data. They closely followed the observed data for the fourth year post-vaccination.

The estimated ICER for rotavirus vaccination was based on the modelled data at the time of vaccine introduction. Our earlier results [18] indicated that the model underestimated the reduction in hospitalisation rates. Our present results show that herd effect on the one hand, and lack of waning on the other, were the main differences between the original static model and real-life data (Figure 3, Tables 3 and 4). Adjusting the model for these factors produced an estimated ICER slightly more favourable to rotavirus vaccination than the estimated ICER without these adjustments (Table 5). The change in the ICER was small (approximately a 10% improvement), because the major impact of change was mainly measured two years after vaccine introduction when the hospitalisation rate was already reduced. The ICER was calculated from the perspective of the healthcare system (direct medical costs only), and so did not capture some categories of cost such as lost productivity from parents taking time off work to look after a sick child. Such costs were not included because we were unable to collect data on them in a real-life setting. A further reduction in the ICER would be expected with an analysis performed from a societal perspective capturing a wider range of costs.

**Table 5:** Cost-effectiveness of rotavirus vaccination pre- and post-adjustment.

	Cost	Difference	QALY	Difference	ICER
**Pre-adjustment**					
No vaccination	70 €		−0.002		
Vaccination	139 €	69 €	−0.00063	0.00138	51 000 € [23]
**Post-adjustment**					
No vaccination	70 €		−0.002		
Vaccination	135 €	65 €	−0.00055	0.00145	44 828 € (−10%)

ICER, Incremental cost-effectiveness ratio; QALY, quality-adjusted life-year.

## Discussion

This analysis compared observed data on rotavirus-related hospitalisations collected in routine clinical practice for four years post-vaccination in Belgium with previously modelled estimates of the effect of vaccination in the same country. The observed reduction in hospitalisations with data from two years post-vaccination has previously been shown to exceed the reduction predicted by the static model [18]. Two differences between the modelled and the observed data were identified that could explain this discrepancy. First, the observed data indicated an indirect herd effect in infants too young (aged <2 months) and too old for vaccination when the vaccine was introduced, which was not included in the model. Second, the model assumed a waning of vaccine efficacy over time based on clinical trial data, which did not appear to be reflected in the observed data from a real-life situation over time frames of three or four years.

Regarding waning of vaccine efficacy, analysis of the vaccine efficacy results of the European trial may offer an explanation that better helps in understanding the difference between the modelled and the observed data. Vaccine efficacy is normally measured as the proportion of one minus the ratio of events that appear in the study arm that received the vaccine divided by the number of events that occur during the same time interval in the non-interventional arm. When analysing the vaccine efficacy in the first and subsequent years of the trial, researchers assume that if a dramatic decrease in events occurs in the non-interventional arm in the second year compared with the first year, as seen in the European trial (the decrease observed in the first versus subsequent year is >40%), a similar decrease should also be observed in the vaccinated arm on top of the measured vaccine benefit of the first year. Any deviation from this result is explained as a reduction in vaccine efficacy called vaccine waning. This assumption is hard to accept as the explanation. The absolute number of events in the vaccinated group during the second year amounted to about the same values as in the first year. So, most of the decrease in vaccine efficacy in the second year in the trial was due to a sharp decrease in the number of events in the denominator, rather than to a sharp increase of the numbers in the vaccinated arm. We hypothesise that the results in the non-interventional arm could have been influenced by a herd effect in the trial, because the randomisation process included 2 vaccinated children for 1 non-vaccinated child. This 2∶1 randomisation may have further decreased the number of events in the non-vaccinated arm in the second year of the trial. As a result of this observed evidence – a large imbalance in the number of events observed over time in the non-vaccinated arm – the true vaccine efficacy measured in the trial may be an underestimate compared with vaccine effectiveness observed in real life, as seen here in the impact study results. It is of interest that the decrease in the subsequent year seen in the 2∶1 randomised trial (>40%) was greater than that observed in a 1∶1 randomised trial of rotavirus vaccination conducted in the US, where the reduction from the first year to the second was approximately 15% [27].

Even if we introduce a correction into the model by excluding the waning scenario (adjusted results in Table 3), the model still underestimates the total vaccine benefit, mainly because of the indirect protection in infants too young to be vaccinated (aged <2 months). This can be seen in Figure 4, where the change in number of hospitalisations between pre-vaccination and the second and fourth years post-vaccination in children aged <1 year was considerably larger in the observed data (Figure 4A) than in the modelled data (Figure 4B). The indirect vaccine efficacy seen in these very young infants is likely to remain at steady-state level. This analysis also provides indirect information about rotavirus transmission in children. Since rotavirus vaccination appeared to have an indirect protective effect on young infants, our results suggest that children in the age range eligible for vaccination can infect younger children.

If vaccination alters the natural transmission of rotavirus in the population outside the at-risk group, it is possible that an age-shift of rotavirus disease could occur, as predicted by dynamic models [7]. However, if the wild-type rotavirus still circulates in the whole population, allowing reinfection and boosting of natural immunity, age-shifts of rotavirus disease may be less likely to happen after introducing vaccination. It is not yet known how rotavirus vaccination will affect rotavirus transmission. It is, however, likely that reported observations over longer time periods will see less important herd effects per year than observed here as soon as the whole at-risk population (children aged <5 years) has been vaccinated.

**Figure 5:**
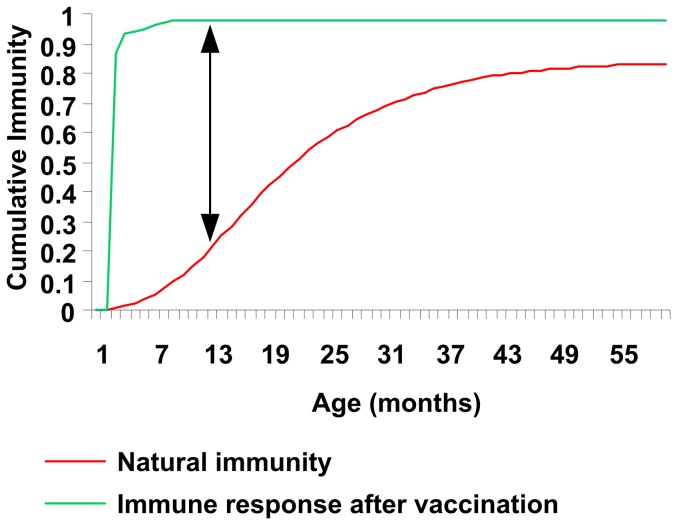
Natural immunity and immune response after vaccination, showing the net effect of vaccination (arrow line).

Our analysis of the observed data suggests that no reduction in vaccine efficacy (vaccine waning) occurred in real life during the first 4 years. It is known that subjects repeatedly exposed to rotavirus gradually build up natural immunity over time. This has been well illustrated by Velazquez and colleagues [28] and others [29]. The observed age-related disease pattern (more cases in young children than in older ones) reflects this immunity build-up, together with other factors that could affect exposure such as behaviour changes. Therefore the effect measured in a clinical trial is not only the vaccination effect, but is a difference between vaccinated and unvaccinated groups (which can be called a net effect) (Figure 5). As natural immunity develops over time in the non-vaccinated group, the net effect would change over time, and that could be mistakenly interpreted as vaccine waning. Herd protection effects could influence the change in net effect as natural immunity would be larger in its absence (because exposure to the virus would be larger).

The results presented in this paper indicate that the ICER estimated from the model for vaccination versus no vaccination, using vaccine efficacy results from randomised controlled trials, may have underestimated the benefit of rotavirus vaccination. Adjusting for that difference would result in a model outcome more closely related to the observed data. The effect is marginal from a healthcare system perspective, as the benefit is mainly seen after two years of vaccine exposure when hospitalisation rates are already low. However, it may have a larger impact on the ICER considered from a societal perspective. We conducted a simulation exercise to explore the potential effect if the reduction in hospitalisations observed in this study were also to occur across the whole disease management area of non-hospital medical visits and indirect costs. If non-hospital medical visits and indirect costs are reduced by the same amount as observed for hospitalisations, the ICER results estimated by the model would improve by >30%. Collecting real-life data on non-hospital medical visits and indirect costs to test this prediction would be a valuable area for future research. This finding will of course be country-specific, depending on the specific disease management programmes in place and whether the economic assessment is conducted after reaching the steady-state level.

In conclusion, it is likely that previously published economic models underestimated the total benefit of rotavirus vaccination, by not including an estimate of herd protection and by including a vaccine waning effect that was not reflected in real-life conditions during the first 4 years of vaccine introduction. These findings could be applicable in other disease areas in which natural immunity develops over time as a result of regular exposure to the infectious agent, although this is not often observed. Static cohort models have major difficulties in capturing such effects and may therefore underestimate the total benefit of vaccines when introduced in children.
